# pH-dependent transcriptional profile changes in iron-deficient *Arabidopsis* roots

**DOI:** 10.1186/s12864-020-07116-6

**Published:** 2020-10-06

**Authors:** Huei-Hsuan Tsai, Wolfgang Schmidt

**Affiliations:** 1 Institute of Plant and Microbial Biology, Academia Sinica, Taipei, 11529 Taiwan; 2 Biotechnology Center, National Chung-Hsing University, Taichung, 40227 Taiwan; 3 Genome and Systems Biology Degree Program, College of Life Science, National Taiwan University, Taipei, 10617 Taiwan

**Keywords:** Ambient pH, Coumarins, Iron deficiency, Iron uptake, RNA-seq, Transcriptome, Alkaline soil

## Abstract

**Background:**

Iron is an essential element for plants and abundantly present in most mineral soils. The mobility of iron is, however, dependent on the redox potential and hydrogen activity (pH) of the soil, factors that may limit its availability to plants in particular at alkaline pHs. Iron deficiency triggers pronounced changes in the transcriptional profile of plants, inducing processes that aid in the acquisition, uptake, and translocation of iron. How ambient pH impact the transcriptional iron deficiency response has not yet been elucidated in detail.

**Results:**

Here, we provide an RNA-seq data set that catalogs global gene expression changes of iron-deficient plants grown at either optimal (5.5) or high (7.0) pH. A suite of 857 genes changed significantly and more than twofold in expression; only 54 genes of this suite were also differentially expressed between iron-deficient and iron-sufficient plants grown at pH 5.5. Among the high pH-responsive genes, 186 were earlier shown to be responsive to short-term transfer to low pH, 91 genes of this subset were anti-directionally regulated by high and low pH. The latter subset contained genes involved in cell wall organization, auxin homeostasis, and potential hubs of yet undefined signaling circuits. Growing iron-deficient plants at high pH also modulated the transcriptional iron deficiency response observed at pH 5.5 by compromising the enzymatic reduction of ferric chelates and favoring the production of iron-mobilizing coumarins.

**Conclusions:**

It is concluded that ambient pH is an important determinant of global gene expression which tunes iron acquisition to the prevailing edaphic conditions.

## Background

Soil pH, i.e. the dynamic equilibrium of H^+^ activity between the soil solution and the negatively charged solid phase, is an important edaphic factor that dictates the availability of mineral nutrients, affects the composition of the microbiome, and determines the composition of plant communities through alterations in the availability of mineral nutrients in the soil [4]. Iron is highly abundant in most soils, but the low mobility of oxidized iron compounds often limits its phyto-availability. In aerated soils, the solubility of iron decreases by a factor of 1000 for each unit increase in pH between 4 and 9, severely restricting the supply of iron at circumneutral or alkaline conditions [16].

In *Arabidopsis* and other non-grass species, iron starvation triggers a sophisticatedly regulated response comprising processes which increase the solubilization of recalcitrant soil iron pools, including the acidification of the rhizosphere, secretion of iron-mobilizing compounds, and reductive splitting of ferric chelates with subsequent release and uptake of Fe^2+^ [11]. Soil pH has a pronounced effect on the acquisition of iron. In acidic soils, high manganese solubility can interfere with iron uptake and may cause secondary iron deficiency [17]. Alkaline conditions, on the other hand, not only restrict the availability of iron, but also compromise the enzymatic reduction of ferric chelates by the plasma membrane-bound reductase FRO2, a central part of the iron uptake mechanism [27]. Restricted mobilization and limited uptake of iron constitute main factors for excluding so-called calcifuge (‘chalk-fleeing’) plants from habitats with alkaline soil reaction [31].

While the transcriptional response of *Arabidopsis* to iron starvation has been well explored (e.g. [10, 23, 28]), most studies were conducted at slightly acidic pH which is optimal for growth, but leaves the influence of alkaline pH on global gene expression profiles undefined. Knowledge on how ambient pH, in particular neutral or alkaline conditions, affects the transcriptional landscape of iron-deficient plants may aid in understanding the bottlenecks of gene regulation in plants that are not well adapted to soils with limited iron availability. Here, we provide an RNA-seq-based inventory of genome-wide gene expression of roots from iron-deficient *Arabidopsis* plants grown either at optimal, slightly acidic (5.5), or high (7.0) pH. To mimic the restricted availability of iron at high pH, we provided an iron source of low solubility to plants grown at neutral pH. Alkaline soil reaction aggravates iron deficiency symptoms, but the effect of pH per se has not yet been clearly distinguished from the response to a lack of iron at a hydrogen activity that is optimal for growth. We believe that the data set provided here allows for gaining valuable insights into the underlying causes of restricted iron uptake, limiting growth in natural or agronomical ecosystems with high pH, and sets the stage for follow up experimentation aimed at identifying novel players involved in the adaptation of plants to the prevailing hydrogen activity.

## Results

### Ambient pH profoundly alters the transcriptomic profile of iron-deficient plants

To investigate the influence of ambient pH on the global gene expression profile of iron-deficient plants, *Arabidopsis* seedlings were grown for 14 days on either optimal (5.5) or high pH (7.0) iron-deficient media, and roots were subjected to transcriptome profiling by RNA-seq. Approximately 24 million reads per treatment were captured during sequencing on the Illumina HiSeq 2000 platform, and mapped to the TAIR10 annotation of the *Arabidopsis* genome.

**Fig. 1:**
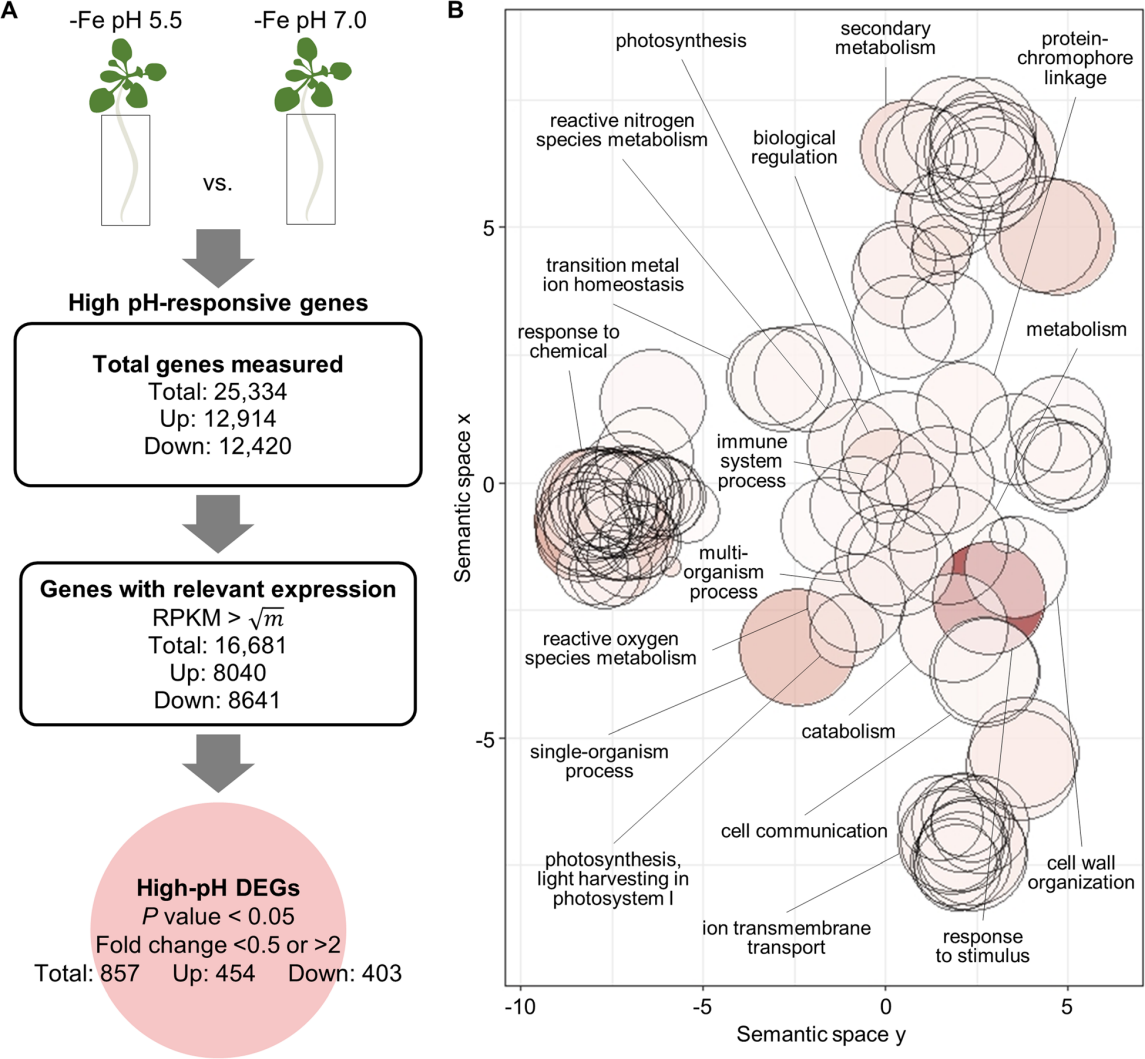
High pH-induced transcriptional changes in iron-deficient plants. **a** flowchart of the experiment that depicts the selection criteria used to identify high-pH DEGs. *m* represents the mean of RPKM values in the whole dataset. **b** GO biological process term analysis result for the 857 high-pH DEGs as summarized by REVIGO. Bubble color indicates the log10 *P* value of enrichment (bubbles of darker colors are more significant) and the size indicates the frequency of the GO term in the underlying GO annotation database (bubbles of more general terms are larger)

A flowchart of the experiment is depicted in Fig. 1a. After filtering lowly expressed genes which may not be of biological relevance (expression level in RPKM < the square root of the mean expression value of the whole dataset), a total of 857 genes were defined as being differentially expressed between iron-deficient plants grown at optimal or high pH media with a < 0.5 or > 2-fold change and a *P* value of < 0.05 (Fig. 1a). The expression of a suite of 37 differentially expressed genes (DEGs) was changed more than tenfold (Supplemental Table 1), indicative of robust alterations in transcriptional activity by a relatively subtle change in ambient pH.

To gain insights into the biological significance of the changes in gene expression, a gene ontology (GO) enrichment analysis for the DEGs was conducted using the Singular Enrichment Analysis (SEA) algorithm available on the AgriGO v2.0 toolkit [29], and visualized with the REVIGO Web server [26]. Categorizing the genes that are responsive to high pH under iron-deficient conditions revealed an overrepresentation of the GO categories ‘response to chemical’ and ‘response to stimulus’, indicating that the majority of pH-responsive genes is involved in adapting the plants to environmental conditions (Fig. 1b). Further, the GO category ‘secondary metabolism’ was significantly enriched. Surprisingly, also genes associated with photosynthesis were overrepresented in the data set, probably mirroring photosynthesis-related processes that are mainly affected in leaves where their expression is altered by high pH to avoid photo-oxidative damage.

Exposure to high pH modulated the iron deficiency response of the plants by altering the expression of several key genes functioning in the acquisition of iron. In high pH plants, *FRO2* transcript levels were reduced by approximately 50% relative to plants grown at pH 5.5 (Supplemental Table 1), matching previous observations of severely compromised in vivo ferric reduction at high pH [27]. By contrast, expression of the 2-oxoglutarate-dependent oxygenase *SCOPOLETIN 8-HYDROXYLASE* (*S8H*) was more than threefold induced when plants were grown on high pH. S8H catalyzes the biosynthesis of the iron-mobilizing coumarin fraxetin (7,8-dihydroxy-6-methoxy-2*H*-chromen-2-one) which is secreted into the rhizosphere under iron-deficient conditions, particularly at high pH [19, 24, 25, 30]. Fraxetin mobilizes iron by both reduction and chelation, and may form stable complexes with Fe^2+^ [19, 30]. Fraxetin-mediated iron reduction is favored by mildly alkaline pH, compensating for the reduced enzymatic ferric reduction activity under such conditions. Interestingly, expression of another iron-responsive gene in the coumarin biosynthesis pathway, the cytochrome P450 *CYP82C4*, was completely abolished when plants were grown on high pH media (Supplemental Table 1). CYP82C4 catalyzes the hydroxylation of fraxetin to form sideretin (5,7,8-trihydroxy-6-methoxy-2*H*-chromen-2-one), a catecholic coumarin with a lower pH optimum for iron mobilization when compared to fraxetin [19]. It thus appears that ambient pH can modulate the activity of metabolic enzymes to prioritize the production of specific compounds in order to adapt iron acquisition to the prevailing edaphic conditions. It is noteworthy that the activity of the two enzymes was altered at the transcriptional level, suggesting that ambient pH is sensed and relayed to the nucleus to modulate transcript abundance, possibly via recruitment of trans-acting factors. Such a putative pH sensing system has not been described in plants previously.

### Ambient pH modulates the iron deficiency response

**Fig. 2:**
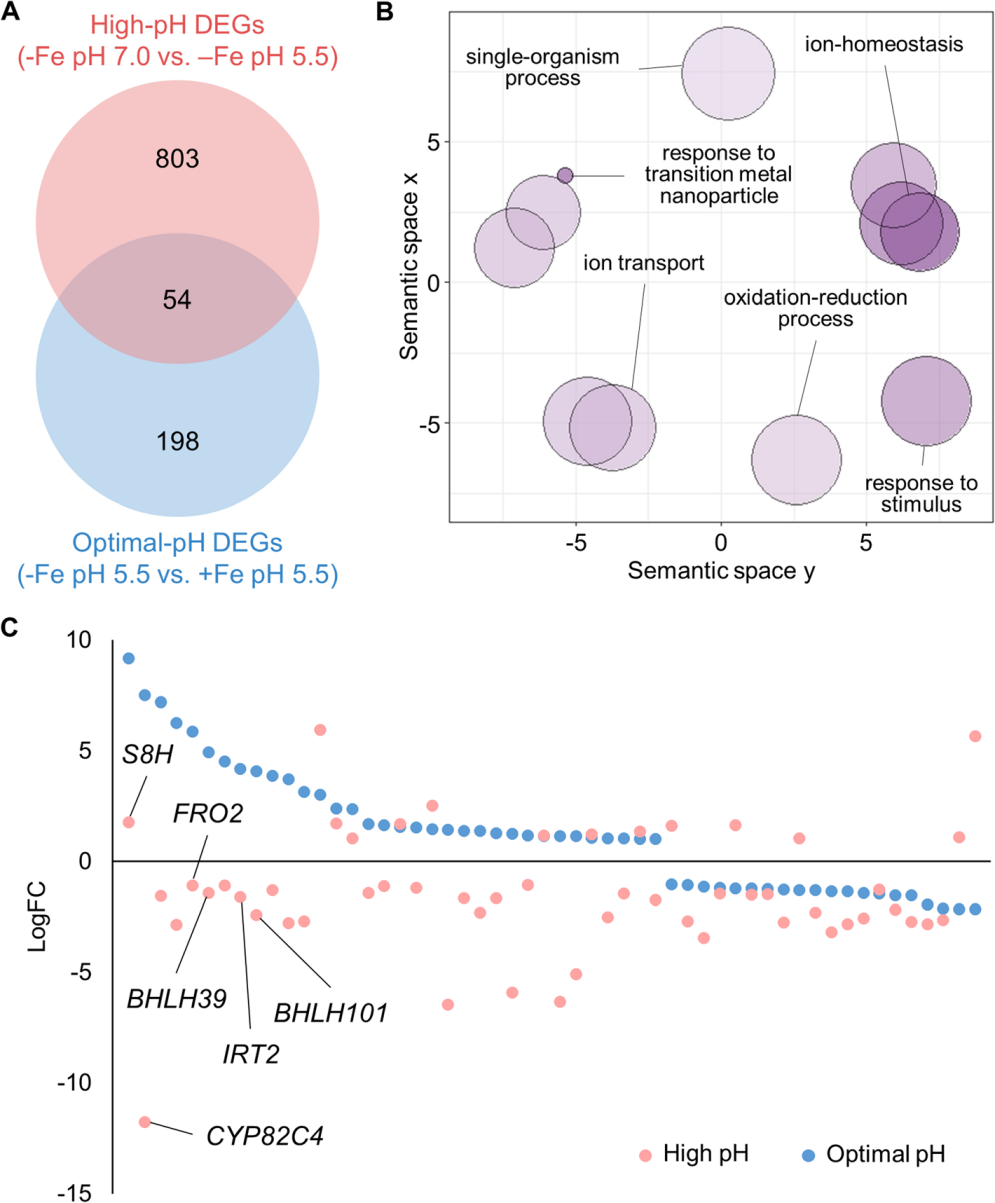
Comparative analysis between high-pH and optimal pH DEGs. **a** Venn diagram comparing the DEGs in high-pH and optimal-pH datasets. **b** GO biological process term analysis result for the common DEGs in (**a**) as summarized by REVIGO. Bubble color indicates the log10 *P* value (bubbles of darker color are more significant) and the size indicates the frequency of the GO term in the underlying GO annotation database (bubbles of more general terms are larger). **c** Visualization of expression changes of the common DEGs in (**a**) in both the high-pH and optimal-pH datasets, where the x-axis represents different genes and the y-axis represents the logFC values. Only relevant genes are labeled; refer to Supplemental Table 2 for the complete gene list. Optimal-pH DEGs are from Rodríguez-Celma et al. [22]

A suite of circa 250 genes that are differentially expressed between iron-sufficient and iron-deficient plants surveyed at optimal pH constitute the ‘ferrome’, a robust shift in the transcriptional profile that induces various iron mobilization strategies and aids in recalibrating cellular iron homeostasis (e.g. [6, 10, 22]). To investigate the effect of a pH shift on iron-deficient plants, a comparative analysis was conducted between high pH-responsive DEGs and DEGs from an RNA-seq-based profiling of iron-sufficient and iron-deficient plants grown under conditions similar to that of the current study except that plants were grown on pH 5.5 media [22]. A comparison of the high pH-responsive DEGs with the ferrome revealed an overlap of 54 genes (Supplemental Table 2; Fig. 2a). Genes from this overlap were mainly enriched in GO terms associated with ‘ion homeostasis’ and ‘response to transition metal nanoparticle’ (Fig. 2b). Interestingly, more than half of the genes are oppositely regulated between plants grown at optimal and high pH. Some 74% of the genes that were highly upregulated under iron-deficient conditions at optimal pH were downregulated when plants were grown on high pH media. This group included several key regulators of iron uptake including the subgroup Ib bHLH proteins *bHLH39* and *bHLH101* [33], suggesting a direct or indirect involvement of these proteins in the pH-dependent regulation of iron uptake (Fig. 2c). This subset also comprises the iron transporter *IRT2* and the oxidoreductase *FRO2*, which catalyzes the reductive splitting of ferric chelates prior to uptake, the rate-limiting step of iron uptake [3, 7, 21, 32]. The reasons for this observation remain to be clarified. It may be speculated that the plant response to high pH interferes at some nodes with that to Fe deficiency, suggestive of a differential prioritization of different iron uptake strategies at different pH conditions.

### Exposure of plants to different media pH uncovers putative nodes in pH signaling

**Fig. 3:**
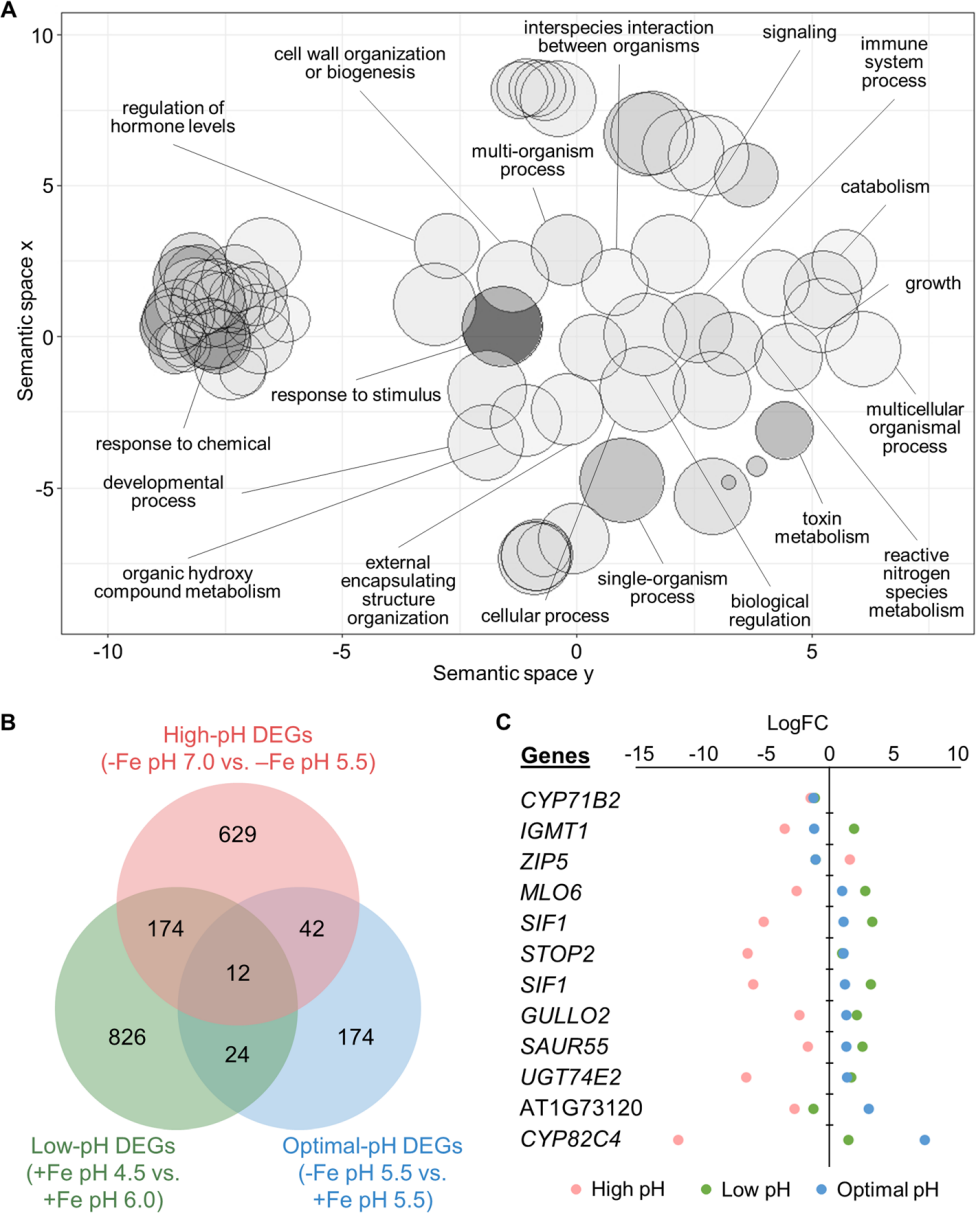
Effect of pH on the transcriptional profile changes in iron-deficient plants. **a** GO biological process term analysis result for the 186 common DEGs between high-pH and low-pH datasets as summarized by REVIGO. Bubble color indicates the log10 *P* value (bubbles of darker color are more significant) and the size indicates the frequency of the GO term in the underlying GO annotation database (bubbles of more general terms are larger). **b** Venn diagram comparing the DEGs in high-pH, optimal-pH, and low-pH datasets. **c** Visualization of expression changes of the 12 common DEGs in (**b**). The x-axis represents the logFC values and the y-axis represents different genes. Low-pH DEGs are from Lager et al. [14]

A previously conducted transcriptional survey of *Arabidopsis* roots exposed to low (4.5) pH medium showed that the expression of a total of 1036 genes was significantly changed after transfer from pH 6.0 [14]. A comparison of this subset to our high pH data set revealed an overlap of 186 genes. In terms of GO categories, these common DEGs are enriched in the biological functions ‘response to chemical’, ‘immune system process’, ‘cell wall organization and biogenesis’, and ‘regulation of hormone levels’ (Fig. 3a). A group of 91 genes were anti-directionally regulated by high and low pH (Table 1). The latter list may contain genes that are involved in the perception and transduction of ambient pH. *PLANT INTRACELLULAR RAS GROUP-RELATED LRR 8* (*PIRL8*), a member of a novel class of plant-specific LRR proteins without clearly defined function [5], was found to be highly upregulated in response to high pH and strongly repressed by low pH (Table 1). Also, several genes involved in cell wall organization such as the pectin lyase At2g43890, the pectin methylesterase *ATPMEI11* and the expansin *EXP17* showed a large high pH /low pH ratio, possibly to compensate compromised cell expansion at alkaline pH. This supposition is supported by a large high pH/low pH ratio of the auxin-related genes *SAUR41* and *SAUR72*, which have been associated with the regulation of cell expansion [13, 18]. High pH /low pH ratios below one were observed for *STRESS INDUCED FACTOR1* (*SIF1*), a root-specific, membrane-anchored LRR kinase with undefined function [33], the aluminum-activated malate transporter *ALMT1*, and the Cys2-His2 zinc-finger domain transcription factor *SENSITIVE TO PROTON RHIZOTOXICITY 2* (*STOP2*)*.* The latter two genes are inducible by low pH stress [14, 12]; almost complete repression was observed under the present (high pH) conditions. Notably, both genes are also induced by phosphate starvation, where ALMT1 and the STOP2 paralog STOP1 repress primary root growth in adaptation to decreased phosphate supply [1, 8]. Also, the auxin efflux carrier family proteins *PILS3* and *PILS5* [2] were strongly down- and highly upregulated by high and low pH, respectively, suggesting that ambient pH alters auxin homeostasis. A low high pH/low pH ratio was also observed for *POLYGALACTURONASE INHIBITING PROTEIN 1* (*PGIP1*). PGIP1 stabilizes the cell wall under acidic conditions and was found to be dependent on STOP1/STOP2 [12]. Notably, *PGIP1*, *STOP2*, *ALMT1*, *SIF1*, and the S-adenosyl-L-methionine-dependent methyltransferases superfamily protein At2g41380 are regulated by STOP1 [12].

**Table 1:** Genes with anti-directional regulation by low and high media pH. High pH data derived from the present study, low pH data are taken from Lager et al. [14]

Locus	Description	High pH	Low pH (1 h)	Low pH (8 h)	High/low pH ratio
		Foldchange			
AT2G41810	Imidazolonepropionase	13.56		0.06	214.45
AT4G01630	EXP17, expansin A17	4.32	0.10		43.85
AT1G43800	SAD6, plant stearoyl-acyl-carrier-protein desaturase family protein	4.26		0.10	42.98
AT1G09750	Eukaryotic aspartyl protease family protein	2.68		0.07	39.09
AT1G33840	Protein of unknown function (DUF567)	10.88	0.28		38.25
AT4G26050	PIRL8, plant intracellular ras group-related LRR 8	8.22	0.23		35.38
AT1G50050	CAP (Cysteine-rich secretory proteins, Antigen 5, and Pathogenesis-related 1 protein) superfamily protein	5.69		0.20	28.49
AT2G43890	Pectin lyase-like superfamily protein	6.32		0.23	26.91
AT5G48430	Eukaryotic aspartyl protease family protein	6.61	0.30		21.76
AT4G33560	WIP5, wound-responsive family protein	4.06		0.27	15.26
AT2G43620	Chitinase family protein	6.43		0.44	14.52
AT1G16510	SAUR41, SAUR-like auxin-responsive protein family	3.94	0.28		14.13
AT5G45340	CYP707A3, cytochrome P450, family 707, subfamily A, polypeptide 3	3.15	0.23		13.62
AT5G60770	NRT2.4, nitrate transporter 2.4	5.60		0.42	13.30
AT3G47380	ATPMEI11, plant invertase/pectin methylesterase inhibitor superfamily protein	4.07		0.31	13.00
AT3G12830	SAUR72, SAUR-like auxin-responsive protein family	3.05	0.33		9.33
AT4G10270	WIP4, WOUND-RESPONSIVE FAMILY PROTEIN	3.41	0.46		7.37
AT5G39890	PCO2, PLANT CYSTEINE OXIDASE 2	2.88		0.43	6.78
AT5G15230	GASA4, GAST1 protein homolog 4	2.98	0.45		6.58
AT1G05300	ZIP5, zinc transporter 5 precursor	3.11	0.50		6.25
AT3G10040	HRA1, HYPOXIA RESPONSE ATTENUATOR1	3.07		0.49	6.19
AT3G27220	Galactose oxidase/kelch repeat superfamily protein	2.77		0.45	6.11
AT5G66460	MAN7, glycosyl hydrolase superfamily protein	2.18		0.43	5.07
AT4G02290	GH9B13, glycosyl hydrolase 9B13	2.35		0.47	4.99
AT1G67750	Pectate lyase family protein	2.31	0.47		4.89
AT1G70710	CEL1, GH9B1, glycosyl hydrolase 9B1	2.24	0.49		4.57
AT4G19230	CYP707A1, cytochrome P450, family 707, subfamily A, polypeptide 1	2.14		0.48	4.44
AT2G29330	TRI, TROPINONE REDUCTASE	0.49		2.28	0.22
AT1G35260	MLP165, MLP-LIKE PROTEIN 165	0.47		2.26	0.21
AT4G38470	Dehydrin family protein	0.47		2.31	0.20
AT5G13750	ZIFL1, ZINC INDUCED FACILITATOR-LIKE 1	0.38		2.05	0.18
AT4G15610	Uncharacterized protein family	0.45		2.45	0.18
AT3G21690	MATE efflux family protein	0.41		2.31	0.18
AT5G66170	STR18, SULFURTRANSFERASE 18	0.41		2.41	0.17
AT1G13600	BZIP58, BASIC LEUCINE-ZIPPER 58	0.45	2.65		0.17
AT3G14990	DJ1A, DJ-1 HOMOLOG A	0.41		2.42	0.17
AT1G65310	XTH17, XYLOGLUCAN ENDOTRANSGLUCOSYLASE/HYDROLASE 17				
	0.49	2.95		0.17	
AT2G32150	Haloacid dehalogenase-like hydrolase (HAD) superfamily protein	0.49		3.13	0.16
AT1G55920	SAT1, SERINE ACETYLTRANSFERASE 1	0.36	2.56		0.14
AT2G39380	EXO70H2, EXOCYST SUBUNIT EXO70 FAMILY PROTEIN H2	0.39		2.78	0.14
AT4G38540	FAD/NAD(P)-binding oxidoreductase family protein	0.40		2.88	0.14
AT3G11340	UGT76B1, UDP-DEPENDENT GLYCOSYLTRANSFERASE 76B1	0.34		2.47	0.14
AT1G53990	GLIP3, GDSL-MOTIF LIPASE 3	0.31		2.27	0.14
AT1G67810	SUFE2, SULFUR E2	0.32	2.35		0.13
AT1G55850	CSLE1, CELLULOSE SYNTHASE LIKE E1	0.42		3.37	0.12
AT5G37260	CIR1, CIRCADIAN 1	0.32	2.63		0.12
AT3G22370	AOX1A, ALTERNATIVE OXIDASE 1A	0.34		2.85	0.12
AT1G01720	ANAC2, ARABIDOPSIS NAC DOMAIN CONTAINING PROTEIN 2	0.46	4.12		0.11
AT4G30670	Putative membrane lipoprotein	0.28		2.65	0.10
AT1G43160	RAP2.6, RELATED TO AP2 6	0.33		3.21	0.10
AT2G29490	GSTU1, GLUTATHIONE S-TRANSFERASE TAU 1	0.24		2.32	0.10
AT1G51420	SPP1, SUCROSE-PHOSPHATASE 1	0.41		4.06	0.10
AT1G49570	Peroxidase superfamily protein	0.43		4.36	0.10
AT1G77450	ANAC032, NAC DOMAIN CONTAINING PROTEIN 32	0.27	2.91		0.09
AT1G73260	KTI1, KUNITZ TRYPSIN INHIBITOR 1	0.33		3.75	0.09
AT1G80240	DGR1, DUF642 L-GALL RESPONSIVE GENE 1	0.25	2.95		0.08
AT3G46230	HSP17.4, HEAT SHOCK PROTEIN 17.4,	0.17		2.03	0.08
AT1G17180	GSTU25, GLUTATHIONE S-TRANSFERASE TAU 25	0.17	2.18		0.08
AT5G24090	CHIA, CHITINASE A	0.29		3.94	0.07
AT3G01420	DOX1, PLANT ALPHA DIOXYGENASE 1	0.25		3.53	0.07
AT5G25460	DGR2, DUF642 L-GALL RESPONSIVE GENE 2	0.38		5.47	0.07
AT4G13180	NAD(P)-binding Rossmann-fold superfamily protein	0.20	3.16		0.06
AT2G29420	ATGSTU7, GLUTATHIONE S-TRANSFERASE TAU 7	0.22		3.64	0.06
AT5G61820	Stress up-regulated Nod 19 protein	0.17		2.96	0.06
AT2G22860	PSK2, PHYTOSULFOKINE 2 PRECURSOR	0.23		4.08	0.06
AT1G76520	PILS3, PIN-LIKES 3	0.14	2.66		0.05
AT5G50760	SAUR55, SMALL AUXIN UPREGULATED RNA 55	0.32		6.13	0.05
AT1G05340	ATHCYSTM1, CYSTEINE-RICH TRANSMEMBRANE MODULE 1	0.40		8.31	0.05
AT2G46750	GULLO2, L -GULONO-1,4-LACTONE (L -GULL) OXIDASE 2	0.20		4.62	0.04
AT1G78340	GSTU22, GLUTATHIONE S-TRANSFERASE TAU 22	0.12	2.72		0.04
AT5G49450	BZIP1, BASIC LEUCINE-ZIPPER 1	0.22		6.01	0.04
AT5G47990	CYP705A5, CYTOCHROME P450, FAMILY 705, SUBFAMILY A, POLYPEPTIDE 5	0.07		2.05	0.04
AT1G02850	BGLU11, BETA GLUCOSIDASE 11	0.30		9.09	0.03
AT2G17500	PILS5, PIN-LIKES 5	0.08		2.45	0.03
AT5G13080	WRKY, WRKY DNA-BINDING PROTEIN 75	0.20		6.49	0.03
AT2G41380	S-adenosyl-L-methionine-dependent methyltransferases superfamily protein	0.06	2.09		0.03
AT3G09270	GSTU8, GLUTATHIONE S-TRANSFERASE TAU 8	0.13		5.50	0.02
AT1G61560	MLO6, MILDEW RESISTANCE LOCUS O 6	0.18		7.34	0.02
AT1G21100	IGMT1, INDOLE GLUCOSINOLATE O-METHYLTRANSFERASE 1	0.09		3.96	0.02
AT4G22610	Bifunctional inhibitor/lipid-transfer protein/seed storage 2S albumin superfamily protein	0.11		5.37	0.02
AT5G48010	ATTHAS1, THALIANOL SYNTHASE, THALIANOL SYNTHASE 1	0.05		3.00	0.02
AT2G15490	UGT73B4, UDP-GLYCOSYLTRANSFERASE 73B4	0.05	3.06		0.02
AT5G06860	PGIP1, POLYGALACTURONASE INHIBITING PROTEIN 1	0.06	4.10		0.01
AT3G13950	Ankyrin	0.06		7.14	0.01
AT1G17170	ATGSTU24, GLUTATHIONE S-TRANSFERASE TAU 24	0.13		18.19	0.01
AT5G22890	STOP2, SENSITIVE TO PROTON RHIZOTOXICITY 2	0.01	2.09		0.01
AT1G05680	UGT74E2, uridine diphosphate glycosyltransferase 74E2	0.01		3.45	0.00
AT1G51840	SIF1, STRESS INDUCED FACTOR 1	0.03		10.81	0.00
AT1G08430	ALMT1, ALUMINUM-ACTIVATED MALATE TRANSPORTER 1	0.03		15.97	0.00
AT1G51830	SIF1, STRESS INDUCED FACTOR 1	0.02		9.67	0.00
AT4G31940	CYP82C4, CYTOCHROME P450, FAMILY 82, SUBFAMILY C, POLYPEPTIDE 4	0.00	2.90		0.00

A subset of 12 genes was responsive to all three conditions under investigation and thus represents genes that are highly responsive to both changes in ambient pH and iron supply (Fig. 3b, c). This subset comprises *CYP82C4* and various regulators such as *SIF1*, *STOP2*, and the FIT-regulated F-box/RNI superfamily protein At1g73120. Notably, *CYP82C4*, which was highly upregulated and downregulated under iron deficiency at optimal and high pH, respectively, was also upregulated at low pH conditions even when iron is sufficient, suggesting that the expression of *CYP82C4* is dictated by external pH independent of the iron status.

## Discussion

Alkaline soils are thought to aggravate iron deficiency by rendering the acquisition of iron pools more difficult due to decreased iron availability. Our data show that a difference in ambient pH from 5.5 to 7.0 is causative for considerable differentiation of the global gene expression profile, which goes well beyond simple exacerbation of the iron deficiency response. A relatively large set of genes is anti-directionally regulated by high and low pH, indicating that ambient pH is translated into transcriptional changes that adapt the plant to the prevailing hydrogen activity, a response that appears to be at least partly elicited by pH per se and independent of alterations in the availability of essential minerals such as phosphate and nitrate or toxic elements such as aluminum. Moreover, the iron deficiency response as such is modulated by alterations in ambient pH. While *FRO2* expression is diminished at circumneutral pH, the production and secretion of iron-mobilizing coumarins is induced by high pH, prioritizing the most effective strategy to mobilize iron from otherwise inaccessible pools.

The transcriptional response of iron-deficient plants grown at high pH also revealed a pronounced overrepresentation of several categories containing genes that orchestrate the defense responses to pathogens, including ERF- and WRKY-type transcription factors, PR proteins, and genes involved in proteolysis, secondary metabolism, and hormone signaling (Supplemental Figure 1). All these responses are much less pronounced in iron-deficient plants grown at optimal pH. Exposure to low pH with sufficient iron supply, on the other hand, elicited a more pronounced pathogen response, indicating pH-dependent prioritization of the responses to pathogen attack and iron starvation. In particular, genes related to signaling were overrepresented under low pH conditions. This survey shows that ambient pH considerably modulates the responses to environmental cues by altering the transcriptional landscape of iron-deficient plants to secure and optimize fitness of the plant under a given set of environmental conditions.

## Conclusions

Our transcriptomic survey confirms previous observations that relatively small changes in media pH can have large impact on gene expression profiles. In addition, we show here that external pH significantly alters the response to iron deficiency by prioritizing specific, pH-dependent modules, allowing for a more efficient iron acquisition which is accurately tuned to a given set of edaphic conditions. Furthermore, the current data shed light on the intertwining of various signaling pathways, where external pH massively influence the decision-making process of plants by modulating the hierarchy of the responses to environmental stimuli.

## Methods

Seeds of the *Arabidopsis* (*Arabidopsis thaliana*) accession Col-0 were obtained from the Arabidopsis Biological Resource Center (Ohio State University). Plants were grown under sterile conditions in a growth chamber on agar-based media as described by Tsai et al. [30]. The growth medium comprised 5 mM KNO_3_, 2 mM MgSO_4_, 2 mM Ca (NO_3_)_2_, 2.5 mM KH_2_PO_4_, 70 μM H_3_BO_3_, 14 μM MnCl_2_, 1 μM ZnSO_4_, 0.5 μM CuSO_4_, 0.01 μM CoCl_2_, and 0.2 μM Na_2_MoO_4_, supplemented with 1.5% (w/v) sucrose, and solidified with 0.4% Gelrite pure (Kelco). For iron-deficient optimal-pH media, 100 μM ferrozine and 1 g/L MES were added, and the pH was adjusted to 5.5 with KOH. For iron-deficient high-pH media, 40 μm FeCl_3_ and 1 g/L MOPS were added, and the pH was adjusted to 7.0 with KOH. Plants were grown on media for 14 d.

For RNA-seq analysis, total RNA was isolated from roots of 14-d-old plants grown under iron-deficient conditions either at optimal (5.5) or high (7.0) pH using the RNeasy Plant Mini Kit (Qiagen). Libraries for RNA-seq were prepared by using the Illumina TruSeq RNA library Prep Kit (RS-122-2001, Illumina) according to the manufacturer’s protocol. Briefly, 4 μg of total RNA per sample were used for library construction. PolyA RNA was captured by oligodT beads and fragmented when eluted. cDNA was synthesized from fragmented RNA using reverse transcriptase (SuperScript III, Cat. No. 18080–093, Invitrogen) and random primers. Reactions were cleaned up with Agencourt AMPure XP beads (Beckman Coulter Genomics). Libraries were end-repaired, adenylated at the 3′ end, ligated with adapters and amplified according to the TruSeq™ RNA Sample Preparation v2 LS protocol. Finally, the products were purified and enriched with 10 cycles of PCR to create the final double-stranded cDNA library. Final libraries were analyzed using Agilent High Sensitivity DNA analysis chip (Cat. no.5067–4626, Agilent) to estimate the quantity and size distribution, and were then quantified by qPCR using the KAPA Library Quantification Kit (KK4824, KAPA). The prepared library was pooled for single-end sequencing using Illumina HiSeq 2500 with 101-bp single-ended sequence read. Transcript abundance was calculated by first mapping reads to the Arabidopsis TAIR10 genome using Bowtie2 [15]. Unmappable reads were mapped to the TAIR 10 genome sequence by BLAT [9]. Read counts were computed using the RackJ package (http://rackj.sourceforge.net/) and normalized using the TMM-quantile method [20]. Normalized read counts were transformed into normalized RPKM values. Z-test was carried out for detecting differentially expressed genes. Two independent biological replicates were performed. RNA-seq data are available at BioProject ID PRJNA664641 (bioprojecthelp@ncbi.nlm.nih.gov).

Gene ontology (GO) enrichment analysis was analyzed using the Singular Enrichment Analysis (SEA) available on the ArgiGO v2.0 toolkit [29]. The analysis was performed using the following parameters: selected species: *Arabidopsis thaliana*; Reference: TAIR genome locus (TAIR10_2017); Statistical test method: Fisher; Multi-test adjustment method: Yekutieli (FDR under dependency); Significance level: 0.05; Minimum number of mapping entries: 5; Gene ontology type: Complete GO. Significantly enriched GO terms were summarized and visualized using REVIGO [26], with a similarity setting of 0.7 and SimRel as the semantic similarity measure. Final figures were plotted in R (version 3.6.2).

## Supplementary information

**Additional file 1: Supplemental Table 1.** High-pH DEGs.

**Additional file 2: Supplemental Table 2.** Common DEGs between high-pH and optimal-pH datasets.

**Additional file 3: Supplemental Figure 1.** MapMan visualization of the biotic stress pathway for the DEGs from different transcriptome datasets. Original figure generated based on the referenced data.

## Data Availability

The RNA-seq datasets are available at NCBI Sequence Read Archive under the accession number PRJNA664641.

## Abbreviations

RNA-seq: RNA-sequencing
GO: Gene onthology
qPCR: Quantitative polymerase chain reaction
RPKM: Reads per kilobase of transcript per million reads mapped
