# Supplementary material for: pH-dependent transcriptional profile changes in iron-deficient Arabidopsis roots
Source: BMC Genomics. 2020 Oct 6;21:694. doi: 10.1186/s12864-020-07116-6 (PMC7539395; doi:10.1186/s12864-020-07116-6)
Supplement: Supplementary file 3 — Additional file 3: Supplemental Figure 1. MapMan visualization of the biotic stress pathway for the DEGs from different transcriptome datasets. Original figure generated based on the referenced data. [file 12864_2020_7116_MOESM3_ESM.pdf]

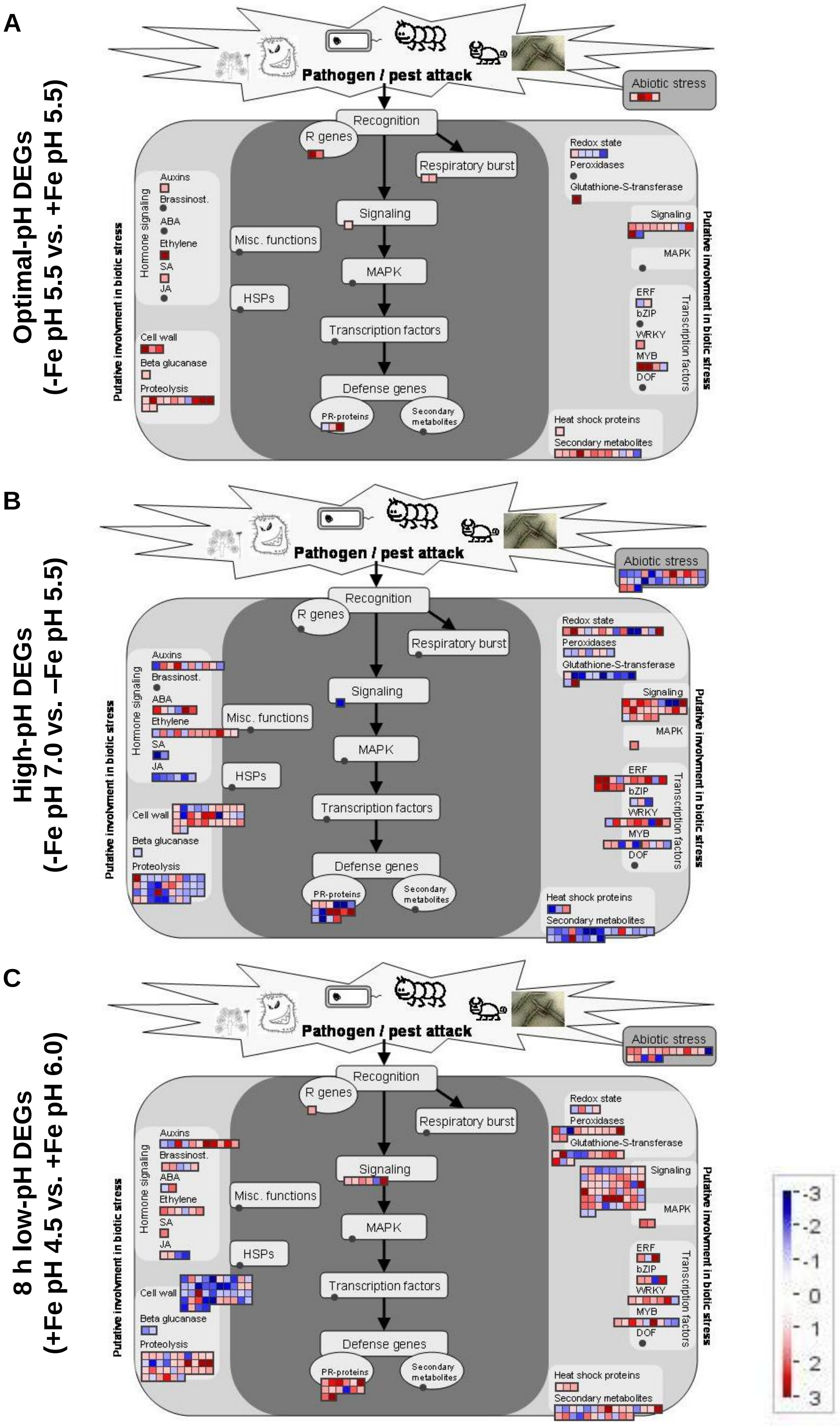

Supplemental Figure 1. MapMan visualization of the biotic stress pathway for the DEGs from different transcriptome datasets. A, Optimal pH DEGs. B, High pH DEGs. C, 8 h low pH DEGs. Red boxes denote up-regulated genes and blue boxes denote down-regulated genes. Optimal-pH DEGs are from Rodríguez-Celma et al. (2013). Low-pH DEGs are from Lager et al. (2013).
